# P370: Antimicrobial copper (Cu+) implementation and its influence to the epidemiological data in elementary school population

**DOI:** 10.1186/2047-2994-2-S1-P370

**Published:** 2013-06-20

**Authors:** P Efstathiou, E Kouskouni, K Karageorgou, M Tseroni, Z Manolidou, S Papanikolaou, E Logothetis, H Tzouma, C Petropoulou, I Agrafa

**Affiliations:** 1 National Health Operations Centre, Athens, Greece; 2 Medical School of the University of Athens, Microbiology laboratory of Aretaieio Hospital, Ministry of Health, Athens, Greece

## Objectives

The aim of this study was to evaluate the epidemiological data in elementary school students after implementing Cu^+^ in multi- touch surfaces.

## Methods

Antimicrobial copper alloy (Cu 63% - Zn 37%, Low Lead) was used to cover or replace multi-touch surfaces (handrails, stair railings), in five elementary schools (N = 1596 students). Epidemiological surveillance of flu-like symptoms was conducted from the 40th week of 2011 to 15th week of 2012 and recorded absenteeism among students based on a specific protocol.

## Results

A significant reduction of pathogenic strains and viruses after the implementation of antimicrobial copper Cu^+^ influenced the occurrence of respiratory infections of viral etiology. A decrease of seasonal influenza (Influenza Like Illness) was recorded on the students of these schools. Clinical morbidity index of students was recorded at 36, 01% (average 5 schools), while in the community the same period (2011-2012) the rate was 48, 8%.

## Conclusion

The use of antimicrobial copper in places with great population concentrations and crowded places such as schools is an innovative application, which in combination with hand hygiene contributes significantly to the reduction of viral respiratory tract infections and emerging as one of the most important allies to the Public Health.

## Disclosure of interest

None declared

